# Neurology

**Published:** 1904-08-27

**Authors:** 


### NEUROLOGY.

**General Paralysis.**—The various types of general paralysis and the difficulties in its diagnosis are discussed in two clinical lectures by Savage.[1] General paralysis is a group of cases that have many similarities, the chief characteristic being a progressive nature that ends in paralysis and death within a comparatively limited time. It is a disease with distinct stages; it is common to have a threatening or warning, followed by an acute period, which is then followed by a paretic or indolent stage, and then comes the last or paralytic stage. Any one of these stages may be arrested for a time. There are many varieties or forms of general paralysis: (1) The acute delirious kind, (2) the ordinary kind, (3) the melancholic type, (4) the progressive demented type, (5) general paralysis of the double form, (6) spinal general paralysis, (7) female general paralytics, (8) the adolescent type, and (9) senile general paralysis. The acute form is very like acute delirious mania, and is apt to be confused with it, unless there be a relapse, when the physical signs of general paralysis will be apparent. Such cases are rapidly fatal. A great characteristic of the ordinary form is that the man loses his highest and last accomplishment; he is not quite able to do what he did best before. He may have a fit or be hysterical, or have a temporary syphilitic lesion—*e.g.* ptosis. A general paralytic sleeps unduly well. Then comes restless irritability and intolerance of restraint, and "facility"—*i.e.* he can be easily led, quite unlike the chronic alcoholic. With this occur physical signs, inequality of pupils, slurring of speech, and a want of facial expression. It is to be noted that if there be exaltation it is a universal and benevolent one. This stage of exaltation may last some three or four months and is followed by a remission, in which the man gradually gets more or less fat and fatuous, and gradually feebler, and finally he passes into the paralytic stage. The hypochondriacal form is very misleading and apt to be mistaken for hypochondriacal melancholia. The characteristic, however, is a want of consistency between the physical and the mental symptoms, *e.g.* a man who appears to be getting miserable and is always complaining about his bowels but who yet is getting fat. A still more difficult form is that of simple dementia; he slowly becomes fat and stuporous. Such cases tend to have fits early, which not unfrequently end the case. Cases of double form are very misleading; they get attacks of exaltation or mania, followed by a remission, and subsequently melancholia, and so on. Spinal cord changes, either of the spastic or locomotor-ataxic type, may precede the cerebral and mental changes. Such cases may not be recognisable as general paralysis until later on. General paralysis is rare in women, who, if affected, very rarely get any ataxic symptoms. Adolescent general paralysis may be mistaken for disseminated sclerosis. Such cases are invariably the subjects of congenital syphilis. There is generally pretty rapid and progressive dementia, with a rapid passage from the first to the second stage, and from that to the final paralytic condition. In the diagnosis of these conditions stress should be laid on the physical signs. Thus, if a man over 40, with a history of syphilis, presents slight mental symptoms, or neurasthenia or hysteria, then the presence of unequal pupils or an alteration in the knee jerks will show that the case is general paralysis. A true case of neurasthenia following, say, influenza, recovers, but may break down again; but, in any case, there is no intellectual defect. On the other hand, a neurasthenic general paralytic will show defects along his special lines. The most common cause of error in diagnosis is alcoholism, which may produce almost exactly the same symptoms; another cause is tumour of the brain and syphilitic brain changes. In this connection it may be remembered that headache and changes in the optic discs are absent in general paralysis; again, cranial nerve implication is most exceptional. Mental changes occurring in a case of locomotor ataxy generally indicate general paralysis, but such patients may become insane quite apart from general paralysis. Such patients, instead of having the general benevolent exaltation, have almost always some type of delusion of persecution. Fits occurring for the first time after 40 years of age generally indicate either general paralysis or a brain tumour. In the case of general paralysis the patient is almost invariably worse in all his symptoms after a fit. The fit does not pass off so cleanly as in epilepsy. On the other hand, the apoplectiform fits which may occur leave him much less damaged than if there had been a hæmorrhage.

**Hemiplegia.**—The general opinion concerning the respiratory movements in hemiplegics is that the two sides of the thorax move equally in ordinary respiration, but if the patient takes a deep breath and brings into action the extraordinary muscles of respiration, the half of the thorax on the paralysed side often expands less than the other. Hughlings Jackson stated, however, in 1895 that in ordinary automatic respiration the amplitude of superior intercostal movements is greater on the paralysed side than on the other, while in volitional movements the usual law of greater action on the sound side would be found. The question has recently been re-investigated by Clark,[2] who found no exceptions to Hughlings Jackson's statements in 161 cases of hemiplegia. The explanation of the phenomenon is that destructive lesions of the internal capsule cut off the cortical inhibitory control over the respiratory centre which therefore overacts on the paralysed side. As regards the cortical representation of respiration, Spencer showed in monkeys that two centres exist—inhibitory and acclerating, the former just outside the olfactory tract, the latter more posterior and external. Vascular lesions would thus be more likely to damage the former centre and its tracts and thus cause the symptom first described by Jackson. These facts show that the intercostals have a double innervation—from the cortex and from the respiratory centre. The principle underlying the respiratory phenomena is of wide application; it may be demonstrated in all muscles bilaterally susceptible of both automatic and volitional movements, *e.g.* in those supplied by the seventh nerve, where a slight movement is more conspicuous on the paralysed side, a strong one less so; it also occurs in the action of the masseters and in the act of swallowing.

**Sensory Tract.**—Several cases of tumour of the brain implicating the sensory tract are described by Hoppe,[3] with the object of throwing light on the cortical localisation of sensory functions. The anatomical facts hitherto gained in reference to the thalamus and cortex and their connections are the following. The optic thalamus is connected by fibres with a large section of the cortex, and reversely the cortex is connected with the thalamus. All the centripetal tracts end in the thalamus; none pass through straight to the cortex. The thalamo-cortical bundles pass through all parts of the posterior limb of the internal capsule, not massed in one bundle like the pyramidal tract, but spread out in a fan-shaped way, and pass to the cortex of the frontal, parietal, occipital, and temporal lobes. The physiological results of experiment are far from being as exact as the anatomical. All experimenters have found hemianopsia resulting from lesions of the thalamus. Some—*e.g.* Ferrier—find additionally sensory disturbance of the opposite side of the body, whilst others do not. The clinical investigations in man are also contradictory—*e.g.* Long finds that thalamic lesions produce permanent hemianæsthesia, whilst others fail to find this. Experiments on the cortex have been made with the view of determining whether any sensory disturbance results. Schäfer, as the result of operating on monkeys, says that a complete voluntary motor paralysis of a part may be produced by cortical lesion without perceptible loss of tactile sensibility, and is therefore not the result of sensory disturbance. On the other hand, Flechsig and Munk think that the sensory and motor areas are identical. Clinical and pathological investigations in man show that acute softening of the cortex of the area supplied by the middle cerebral artery produces, in addition to a complete hemiplegia, a complete hemianæsthesia. Chronic degeneration and atrophy of the Rolandic area, produced likewise by thrombosis of a branch of the middle cerebral artery, was not followed by a permanent loss of sensation of the opposite side of face and arm. Tumours destroying the cortex and subcortical regions of the motor area very frequently have subjective disturbances of sensation such as pain, formication and feeling of numbness, and slight objective disturbances may occur. Acute lesions, both of the thalamus and corona radiata, confirm the newer anatomical discoveries of the thalamus and thalamo-cortical fibres as the sensory conduction tract. On the other hand, chronic lesions of the thalamus, subcortical white matter and cortex, may exist without any pronounced disturbances of sensation. These latter leave us with but one explanation—that general body sensations have a bilateral representation, and that sensory impressions may reach the cortex by vicarious routes if the paths which they usually follow happen to be destroyed.

**Prognosis in Tabes.**—Collins[4] classifies the symptoms of tabes into (1) the preataxic stage, (2) the ataxic stage, and (3) the profoundly inco-ordinate and hypotonic stage. The duration of the first stage in 140 cases was on the average three years and five months. The factors that influence the prognosis are, (1) the clinical type of the disease, (2) anatomical type, *i.e.* whether cervical or lumbar, (3) sex and age, (4) the individual, his occupation, and the treatment. The prognosis is most favourable in the cases of a motor type, especially since the introduction of the Fraenkel system of exercises. It is unfavourable when severe disturbance of the urogenital system and trophic symptoms are early and conspicuous manifestations. The prognosis is worse in cases of sudden onset, better when this is slow or insidious. Cervical and cervico-bulbar tabes has a serious prognosis, also early involvement of the nerve supply of the larynx. The course of the disease is more rapid in women, in young tabetics under 35 years, and in those whose occupation necessitates standing or fatiguing use of the legs. The pronounced cases seen 25 years ago are seldom seen to day, probably due to improved methods of treatment. According to Faure,[5] in 60 per cent. of the cases, no matter what the treatment, tabes is arrested in the early stages, or its evolution is so slow that its effect upon the duration of life is very slight. In but 30 per cent. does the disease progress so rapidly as to justify the grave prognosis formerly given to this condition. Five per cent. are clinically cured, whilst a like number progress rapidly and fatally, and their course is marked by fever and symptoms of infection, and lasts but a few months or at most two years.

[1] Clin. Jour., March 9 and 16. [2] Amer. Jour. Med. Sc., Dec. [3] Jour. of Nerv. and Ment. Dis., May. [4] Med. News, Aug. 29. [5] Jour. de Med. de Bordeaux, Aug. 16.

